# Knowledge and practice attitudes regarding the relationship between diabetes and periodontitis: a survey among Swiss endocrinologists and general physicians

**DOI:** 10.1186/s12875-023-02184-5

**Published:** 2023-11-13

**Authors:** Natalia Chatzaki, Alkisti Zekeridou, Elsa Paroz, Giacomo Gastaldi, Catherine Giannopoulou

**Affiliations:** 1 Division of Regenerative Dental Medicine and Periodontology, University Clinics of Dental Medicine, Faculty of Medicine, University of Geneva, 1 Rue Michel-Servet, Geneva, 1211 Switzerland; 2 Faculty of Medicine, University Clinics of Dental Medicine, University of Geneva, 1 Rue Michel-Servet, Geneva, 1211 Switzerland; 3 Diabetology Unit, University Hospitals of Geneva, Geneva, Switzerland

**Keywords:** Periodontitis, Diabetes, Survey, General health physicians, Knowledge and practices, Health education

## Abstract

**Background:**

The objective of the present survey is to assess the knowledge about the relationship between oral health and diabetes and to identify the practice behaviors of Swiss endocrinologists and general practitioners regarding oral health in diabetic patients.

**Methods:**

A thirty- item questionnaire was mailed to 428 internists and 99 endocrinologists working in the French speaking part of Switzerland. Participants were asked about their awareness of the relationship between diabetes and periodontal disease, their practice behaviors as well as their willingness for an interdisciplinary education and collaboration with oral health professionals. The questions were answered according to a three-point or five-point Likert scale.

**Results:**

The response rate was 23%. All participants were aware of the inflammatory and infectious nature of periodontal disease. They all agreed that good periodontal health is important for overall health. However, most of the practitioners responded that only rarely received information during their education curricula on the link between systemic and oral health or concerning periodontal problems in diabetic patients (60.9% for endocrinologists and 54.1% for general physicians); thus, only a minority of health practitioners addresses oral health care to their patients (13% and 15.3%, respectively). Both endocrinologists and general health physicians agreed that an oral health screening could be included in their practice (79% for both groups).

**Conclusions:**

An interdisciplinary education and collaboration among medical and dental health providers should be established to effectively prevent, manage, and control both diabetes and periodontal disease in diabetic patients.

## Background

Periodontal disease is an inflammatory disease initiated by the formation of mixed biofilms on teeth and gingival tissues. Periodontitis is proceeded by gingivitis, which is a superficial inflammation of the gums presenting symptoms such as bleeding, spontaneous or while brushing, and oedema. Gingivitis is reversible; when oral hygiene is well-performed and bacterial deposits are eliminated, the inflammation decreases and gingival health is restored [1]. However, if left untreated in susceptible individuals, gingivitis may progress to periodontitis resulting to a more advanced inflammation that leads in loss of the tooth-supporting tissues i.e. gingiva, bone, periodontal ligament, and can eventually cause tooth loss [2].

Disease occurs when the balance between the microbial biofilm and the host is lost, due to dysbiosis or immune overreaction of the host to the microbial challenge. Several local, systemic, environmental and genetic factors can modify the reactions between bacteria and the host and influence the progression and the severity of the disease [3]. According to the World Health Organization (WHO), periodontitis is a non-communicable disease (NCD) and is classified as the sixth most common human disease. The disease has a prevalence of 45%–50% overall, with the severe form affecting 11.2% of the general population [4]. Recently, a new classification for periodontal diseases and conditions was introduced by the American Academy of Periodontology and the European Federation of Periodontology (AAP/EFP). According to the new case definition, the prevalence of periodontitis was reported to be even higher, at least in  the adolescent cohort (75.6%) [5].

Today, there is a significant body of evidence to support independent associations between severe periodontitis and several NCDs such as diabetes mellitus [6], cardiovascular disease [7], chronic obstructive pulmonary disease [8] and chronic kidney disease [9]. As supported by a plethora of studies, the relationship between diabetes and periodontal disease is bi-directional: diabetes is a primary risk factor for periodontitis and periodontitis is considered to be the sixth complication of diabetes [10]. Dental professionals are often concerned by the diabetic status of their patients since diabetes is an important modifying factor for periodontitis [11]. Non-controlled diabetic patients present a more severe inflammatory response resulting to pronounced clinical and radiographic signs of periodontitis [12]. It is important to mention that among diabetic patients, those with poor glycemic control are at higher risk of presenting more severe periodontal destruction [13] due to the formation of gingival advanced glycation end-products [14] and the uncoupling of vascular endothelial growth factor (VEGF) and inducible nitric oxide synthase (iNOS) expression in the periodontium [15]. Thus, in the assessment of the prognosis of periodontal disease the level of glycemic control is taken into consideration [16].

Two dental parameters, the number of missing teeth and the number of teeth presenting severe periodontal destruction were able to identify cases with unrecognized dysglycemia suggesting that simple periodontal parameters can contribute to identify patients with underdiagnosed prediabetes or diabetes [17, 18]. Furthermore, several clinical studies have shown that periodontal treatment is associated with reductions in hemoglobin A1C (HbA1C) levels of 0.27–0.48% at least 3 months after treatment [19]. Recently, an update of the Cochrane Database Systematic Review first published in 2010 and then in 2015 was conducted, on the effect of periodontal treatment on glycemic control in people with diabetes and periodontitis. Based on 35 randomized clinical trials and 3249 participants, the authors reported an absolute reduction in HbA1c of 0.50% 12 months after periodontal treatment using subgingival instrumentation [20].

This may have an impact not only on the course of diabetes but also on the economic burden imposed by the disease on patients and the health system [21].

There are two main mechanisms linking the oral infection with diabetes and other systemic pathologies: the dissemination of periodontal bacteria and their products to distant sites via the blood stream (bacteremia), and the metastatic spread of the local inflammation [22].

Despite the strong body of evidence supporting the relationship between periodontitis and diabetes, several surveys have shown that oral disease awareness among diabetic patients is low [23–25]. A recent systematic review based on 28 studies revealed that overall, people with diabetes rarely receive oral health education and dental referrals from their care providers [26, 27]. These results reveal the necessity for a multidisciplinary approach involving the patient, the oral health professional, and the diabetes care provider.

The aim of the present survey is to evaluate the knowledge and practice behaviors among a sample of Swiss medical practitioners regarding the relationship between diabetes and periodontal disease.

## Methods

### Sample and data collection

 A cross-sectional survey was carried out involving 428 general health practitioners and a sample of 99 endocrinologists practicing in the French speaking part of Switzerland. An electronic version of a questionnaire together with an information sheet was e-mailed to registered physicians working in private or public and hospital clinics. Both general and specialist practitioners were enrolled. The questionnaire was e-mailed again three months later to obtain a larger number of answers.

The questionnaire consisted of 30 items, some of them adapted from existing questionnaires used in similar studies and was divided in 5 sections [28–32]. The first section concerned personal data such as age, sex, practice location, years since the specialization, university by which the degree was granted and the number of working hours per week. The second part consisted of 8 questions on the knowledge regarding periodontal disease and its link to diabetes. The third part consisted of 7 questions regarding the practice behavior and opinions of the practitioners towards oral health. The last 2 sections consisted of 8 questions on physician’s education concerning the impact of oral health on systemic heath and on his/her willingness for an interdisciplinary education and collaboration. Items were answered according to a three to five-point Likert scale.

The questionnaire was developed based on evidence from the literature and was pilot tested by a group of general practitioners (*N* = 6). The answers and comments from this group were evaluated by a panel of dental health professionals who are familiar with studies pertaining DM and periodontitis. Modifications were made to improve clarity, when necessary.

### Statistical analysis

We used descriptive statistics with frequency and percentages to explore the various items addressed to the dentists. To compare the distribution of responses between the endocrinologists and general physicians on each item, chi-squared tests, or Fisher’s exact tests when appropriate, were conducted. *P*-values below 0.05 were considered statistically significant.

## Results

### Demographics

A total of 527 questionnaires was sent between April 2022 and October 2022. We received 23 answers from the endocrinologists’ group (response rate 23.2%) and 98 answers by the general physicians’ group (response rate 23%) with a total response rate of approximately 23%. Responses were received from 50.5% (*N* = 61) male and 49.5% (*N* = 60) female doctors. The mean age of the participants was 48 years (range 29–70). As shown in Table 1, the years of experience since the postgraduate qualification, varied from 1 to 43 years. 85.1% of the participants had graduated from a Swiss university and 13.2% from other universities; the remaining practitioners did not report the country of their medical diploma. 42.1% of the participants reported working 38 h per week or more, followed by 36.4% who reported working between 25–38 h per week. Most of the endocrinologists work in a hospital (43.5%) whereas the generalists have their own private practice (86.7%).

**Table 1:** Demographics of survey responders (numbers in parenthesis are percentages)

Demographics	Endocrinologists *n* = 23	General health physicians *n* = 98	Total *n* = 121
**Gender**			
Female	15 (65.2%)	45 (45.9%)	60 (49.5%)
Male	8 (34.8%)	53 (54.1%)	61 (50.5%)
**Age** (range in years)	29–62	34–70	
**Years of experience since speciality degree**	1–32	1–43	
**Graduation from university in**			
Switzerland	17 (73.9%)	86 (87.8%)	103 (85.1%)
Other countries	5 (21.7%)	11 (11.2%)	16 (13.2%)
No answer	1 (4.4%)	1 (1%)	2 (1.7%)
**Hours worked per week**			
12–24	2 (8.7%)	22 (22.5%)	24 (19.8%)
25–38	10 (43.5%)	34 (34.7%)	44 (36.4%)
> 38	10 (43.5%)	41 (41.8%)	51 (42.1%)
No answer	1 (4.3%)	1 (1%)	2 (1.7%)
**Type of practice**			
Hospital	10 (43.5%)	3 (3.1%)	13 (10.7%)
Private practice	7 (30.4%)	85 (86.7%)	92 (76%)
Both	5 (21.7%)	9 (9.2%)	14 (11.6%)
No answer	1 (4.4%)	1 (1%)	2 (1.7%)
**Region of practice**			
Urban	20 (86.9%)	64 (65.3%)	84 (69.4%)
Rural	2 (8.7%)	34 (34.7%)	36 (29.8%)
No answer	1 (4.4%)	0	1 (0.8%)

### Evaluation of periodontal knowledge and link with diabetes

As shown in Table 2, all responders (100%) are aware of the inflammatory and infectious nature of periodontal disease. A high proportion of both diabetologists and generalists correctly identify the clinical signs of periodontal disease, such as gingival bleeding (95.9%) and tooth mobility (74.4%). More than two thirds in both groups also report that pain is a clinical sign of periodontal disease. All participants agree that oral health plays an important role on the overall health; however, uncertainty is expressed on the negative effect of periodontitis on diabetes’s control and on the positive effect of periodontal treatment on glycemic control.

**Table 2:** Knowledge regarding periodontal disease and the link between periodontitis and diabetes

Questions	Endocrinologists (*n* = 23)	General health physicians (*n* = 98)	*P value*	Total (*n* = 121)
**Definition of periodontal disease ** ***(multiple answers)***				
Inflammatory and multi-infectious process	23 (100%)	98 (100%)		121 (100%)
Degenerative process	3 (3%)	8 (8.2%)	0.436	11 (9%)
Auto-immune process	0	2 (2%)	0.999	2 (1.6%)
Osteoporosis	0	0		0
Mono-infection	1 (4.4%)	0	0.190	1 (0.8%)
**Which are the main clinical signs associated with periodontal disease? ** ***(multiple answers)***				
Gingival bleeding	22 (95.7%)	94 (95.9%)	0.999	116 (95.9%)
Pain	16 (69.6%)	72(73.5%)	0.906	88 (72.7%)
Tooth mobility	16 (69.6%)	74 (75.5%)	0.747	90 (74.4%)
Alveolar bone destruction	10 (43.5%)	29 (29.6%)	0.301	39 (32.2%)
Caries	1 (4.4%)	12 (12.2%)	0.458	13 (10.7%)
Tooth loss	15 (65.2%)	57 (58.2%)	0.701	72 (59.6%)
**Good periodontal health is important to overall health**				
Agree	22 (95.6%)	97 (99%)	0.312	119 (98.4%)
Disagree	0	0		0
Unsure	0	1 (1%)		1 (0.8%)
No answer	1 (4.4%)	0		1 (0.8%)
**Periodontitis can negatively affect the control of glucose levels in diabetic patients**				
Agree	16 (69.6%)	65 (66.3%)	0.076	81 (66.9%)
Disagree	2 (8.7%)	1 (1%)		3 (2.5%)
Unsure	5 (21.7%)	32 (32.7%)		37 (30.6%)
No answer	0	0		0
**Diabetics with poor metabolic control are at higher risk to develop severe periodontal disease**				
Agree	20 (86.9%)	80 (81.6%)	0.586	100 (82.6%)
Disagree	1 (4.4%)	2 (2%)		3 (2.5%)
Unsure	2 (8.7%)	16 (16.4%)		18 (14.9%)
No answer	0	0		0
**Periodontal treatment may improve glycaemic control**				
Agree	11 (47.8%)	45 (45.9%)	0.928	56 (46.3%)
Disagree	1 (4.4%)	6 (6.1%)		7 (5.7%)
Unsure	11 (47.8%)	47 (48%)		59 (48.8%)
No answer	0	0		0
**It is recommended that physicians should ask their patients if they have regular dental check-ups**				
Agree	19 (82.6%)	84 (85.7%)	0.425	103 (85.1%)
Disagree	1 (4.4%)	3 (3.1%)		4 (3.3%)
Unsure	3 (13%)	9 (9.2%)		12 (9.9%)
No answer	0	2 (2%)		2 (1.7%)
**Patients with poor glycaemic control should have more frequent dental checkups and more frequent scaling**				
Agree	20 (86.9%)	77 (78.6%)	0.042^*^	97 (80.2%)
Disagree	2 (8.7%)	2 (2%)		4 (3.3%)
Unsure	1 (4.4%)	19 (19.4%)		20 (16.5%)
No answer	0	0		0

*P* values were calculated to compare the distribution of responses between the endocrinologists and general physicians

^*^ *P*-values below 0.05 were considered statistically significant

### Practice behaviors

The practice behaviors of the endocrinologists and general physicians and the difference in the distribution of their responses is shown in Table 3. Most of both endocrinologists and general health physicians reported that they only “sometimes” refer their patients to the dentist. On the opposite, 78.3% of the endocrinologists have never been -or rarely been- contacted by a dentist for a diabetic evaluation of a patient. For general health practitioners, this was the case for approximately 46% of them (*p* = 0.001). Some of the practitioners ask their patients questions about their oral health, but only rarely a patient reports pain or discomfort in the oral area during consultation, mainly to the endocrinologist group (*p* = 0.004). Some of the endocrinologists and general practitioners tend to explain on regular basis to their patients the relationship between diabetes and periodontitis (34.8% and 42.9% respectively). All participants agree that health insurance companies should reimburse the cost of periodontal treatment in diabetic patients and that an interdisciplinary collaboration should be established between general heath and oral health practitioners.

**Table 3:** Practice behaviors and opinions regarding oral health and the link with diabetes (never- very often and I do not agree, I agree)

Questions	Endocrinologists (*n* = 23)	Genaral health physicians (*n* = 98)	*P* value	Total (*n* = 121)
**Do you refer your patients to a dentist?**				
Never	1 (4.4%)	2 (2%)	0.552	3 (2.5%)
Rarely	7 (30.4%)	20 (20.4%)		27 (22.3%)
Sometimes	10 (43.5%)	40 (40.8%)		50 (41.3%)
Often	3 (13%)	24 ( 24.5%)		27 (22.3%)
Always	2 (8.7%)	11 (11.3%)		13 (10.8%)
No answer	0	1 (1%)		1 (0.8%)
**During your career, have you ever been approached by a dentist about one of your patients?**				
Never	14 (60.9%)	16 (16.3%)	0.001^*^	30 (24.8%)
Rarely	4 (17.4%)	29 (29.6%)		33 (27.3%)
Sometimes	5 (21.7%)	45 (46%)		50 (41.3%)
Often	0	7 (7.1%)		7 (5.8%)
Always	0	1 (1%)		1 (0.8%)
No answer	0	0		0
**During your consultation do you ask your patients questions related to oral health?**				
Never	3 (13%)	4 (4.1%)	0.038^*^	7 (5.8%)
Rarely	9 (39.2%)	18 (18.4%)		27 (22.3%)
Sometimes	8 (34.8%)	50 (51%)		58 (48%)
Often	3 (13%)	15 (15.3%)		18 (14.9%)
Always	0	11 (11.2%)		11 (9%)
No answer	0	0		0
**Do your patients report any pain or discomfort in the oral area during their consultation?**				
Never	2 (8.7%)	0	0.004^*^	2 (1.7%)
Rarely	8 (34.8%)	13 (13.3%)		21 (17.4%)
Sometimes	12 (52.1%)	62 (63.3%)		74 (61.1%)
Often	1 (4.4%)	19 (19.4%)		20 (16.5%)
Always	0	4 (4%)		4 (3.3%)
No answer	0	0		0
**Do you ever explain to your patients the relationship between diabetes and periodontitis?**				
Never	6 (26.1%)	24 (24.5%)	0.029^*^	30 (24.8%)
Rarely	7 (30.4%)	29 (29.6%)		36 (29.8%)
Sometimes	2 (8.7%)	32 (32.7%)		34 (28.1%)
Often	6 (26.1%)	10 (10.2%)		16 (13.2%)
Always	2 (8.7%)	2 (2%)		4 (3.3%)
No answer	0	1 (1%)		1 (0.8%)
**In France, health insurance companies reimburse the cost of periodontal disease treatment in diabetics. Do you think this procedure would be beneficial in Switzerland?**				
Strongly Disagree	0	3 (3.1%)	0.029^*^	3 (2.5%)
Disagree	0	0		0
Undecided	1 (4.4%)	16 (16.3%)		17 (14.1%)
Agree	3 (13%)	32 (32.7%)		35 (28.9%)
Strongly Agree	19 (82.6%)	46 (46.9%)		65 (53.7%)
No answer	0	1 (1%)		1 (0.8%)
**The management of diabetic patients would be improved if there was interdisciplinary collaboration between general practitioners or diabetologists and the dentists**				
Strongly Disagree	0	0	0.545	0
Disagree	0	4 (4.1%)		4 (3.3%)
Undecided	4 (17.4%)	18 (18.4%)		22 (18.2%)
Agree	10 (43.5%)	51 (52%)		61 (50.4%)
Strongly Agree	9 (39.1%)	24 (24.5%)		33 (27.3%)
No answer	0	1 (1%)		1 (0.8%)

*P* values were calculated to compare the distribution of responses between the endocrinologists and general physicians

^*^ *P*-values below 0.05 were considered statistically significant

### Physicians’ education on the link between oral and systemic health

As shown in Table 4, many of the practitioners did not receive any information on the link between systemic and oral health and/or periodontal problems in diabetic patients, during their education curricula. They all agree on the importance of the topic and that they could consider an oral health screening to be included in their practice (Table 5).

**Table 4:** Physician’s education concerning the impact of oral health on systemic health

Questions	Endocrinologists (*n* = 23)	Genaral health physicians (*n* = 98)	*P value*	Total (*n* = 121)
**Did you receive any education on oral health during your studies?**				
Never	6 (26.1%)	28 (28.6%)	0.965	34 (28.1%)
Rarely	14 (60.9%)	53 (54.1%)		67 (55.3%)
Sometimes	3 (13%)	13 (13.3%)		16 (13.2%)
Often	0	2 (2%)		2 (1.7%)
Always	0	0		0
No answer	0	2 (2%)		2 (1.7%)
**Were you made aware of periodontal problems in diabetic patients during your training?**				
Never	4 (17.4%)	42 (42.9%)	0.046^*^	46 (38%)
Rarely	12 (52.1%)	40 (40.8%)		52 (42.9%)
Sometimes	6 (26.1%)	13 (13.3%)		19 (15.7%)
Often	1 (4.4%)	1 (1%)		2 (1.7%)
Always	0	0		0
No answer	0	2 (2%)		2 (1.7%)
**What other sources of information regarding the link between diabetes and periodontal disease have you received? ** ***(multiple answers)***				
Media	1 (4.3%)	9 (9.2%)	0.685	10 (8.3%)
Medical literature	7 (30.4%)	29 (29.6%)	0.999	36 (29.8%)
Clinical experience	9 (39.1%)	36 (36.7%)	0.999	45 (37.2%)
Continuing education/conferences	7 (30.4%)	40 (40.8%)	0.495	47 (38.8%)
None of the above	10 (43.5%)	35 (35.7%)	0.650	45 (37.2%)

*P* values were calculated to compare the distribution of responses between the endocrinologists and general physicians

^*^ *P*-values below 0.05 were considered statistically significant

**Table 5:** Physicians’ willingness for an interdisciplinary education and collaboration fo the diabetic patient

Questions	Endocrinologists (*n* = 23)	Genaral health physicians (*n* = 98)	*P* value	Total (*n* = 121)
**It would be important to make doctors aware during their training of the relationship between diabetes mellitus and periodontal disease**				
Agree	21 (91.3%)	93 (94.9%)	0.426	114 (94.2%)
Disagree	0	1 (1%)		1 (0.8%)
Unsure	2 (8.7%)	4 (4.1%)		6 (5%)
No answer	0	0		0
**Doctors should be made aware of the symptoms of periodontal disease**				
Agree	21 (91.3%)	93 (94.9%)	0.707	114 (94.2%)
Disagree	0	1 (1%)		1 (0.8%)
Unsure	2 (8.7%)	4 (4.1%)		6 (5%)
No answer	0	0		0
**I need more information on periodontal disease and its impact on diabetes**				
Agree	21 (91.2%)	88 (89.8%)	0.913	109 (90%)
Disagree	1 (4.4%)	5 (5.1%)		6 (5%)
Unsure	1 (4.4%)	5 (5.1%)		6 (5%)
No answer	0	0		0
**Doctors should be trained to screen their diabetic patients for periodontal disease**				
Agree	17 (74%)	83 (84.7%)	0.107	100 (82.6%)
Disagree	3 (13%)	3 (3.1%)		6 (5%)
Unsure	3 (13%)	11 (11.2%)		14 (11.6%)
No answer	0	1 (1%)		1 (0.8%)
**I want to include oral health screening in my practice**				
Agree	18 (78.3%)	78 (79.6%)	0.841	96 (79.3%)
Disagree	2 (8.7%)	5 (5.1%)		7 (5.8%)
Unsure	3 (13%)	15 (15.3%)		18 (14.9%)
No answer	0	0		0

*P* values were calculated to compare the distribution of responses between the endocrinologists and general physicians

## Discussion

The aim of the present cross- sectional survey was to explore the knowledge and attitudes of a sample of Swiss general health physicians and diabetologists regarding the link between periodontitis and diabetes. The questionnaire was sent in practitioners working in the French speaking part of Switzerland. The response rate, even after repeated reminders, was approximately 23%, that is relatively low, but could be explained by the increased hours and workload of the practitioners [33].

The need for more education and for an interdisciplinary approach for diabetic patients was reported in various surveys addressed to endocrinologists, general health practitioners, oral health professionals and diabetic patients [28–31, 34]. The scoping review of Poudel et al. 2017 [26, 27] clearly showed that most diabetic care providers do not address oral health care; main barriers are time constraints and limited knowledge on oral health evaluation. The same limitations were also reported in the present survey. In addition, another obstacle that decreases the interdisciplinary approach as reported by many practitioners, was the non-reimbursement of dental costs by the insurances. A change in the insurance policy of diabetics might ameliorate oral care, particularly in the disadvantaged population. However, a study from Sweden reported that more than one third of people with diabetes did not visit the dentist, despite being entitled for subsidized dental care [35]. Similarly, a national oral health survey in Australia reported that there is no significant difference in dental visits between adults eligible for public dental services and those that are not [32, 36]. Recently, the French health insurance decided to entirely support the cost of periodontal treatment for diabetic patients. For the moment, the efficiency of this governmental package seems to be limited. According to the results of 28 studies in 14 different countries, including 27,894 diabetic patients, analyzed in a systematic review, people with diabetes had inadequate oral health knowledge, poor oral health attitudes and fewer dental visits [26]. In Switzerland, basic health insurance (LAMal) reimburses dental treatment only if it is the result of a serious illness or accident. Insurance under the LAMal does not cover any routine dental treatment. This can be covered only by supplementary dental insurance.

Several surveys have been conducted to explore the knowledge of health providers on the role of periodontitis on systemic health. For exemple, in a cross-sectional study performed on french gynecologists and obstetricians, 88% were aware that periodontitis is an inflammatory disease that can negatively affect pregnancy outcomes. Half of them adressed this issue, with however, only one third of them systemically referring their patients to a dentist. The authors concluded that although periodontal knowledge among french gynecologists/obstetricians was satisfying, their clinical behavior and attitude did not correlate with their knowledge [37]. The same results were depicted in the present survey. Responders were aware of the inflammatory and infectious nature of periodontitis and that diabetic patients should have frequent dental checkups, especially these with poor glycaemic control, but only few of the participants explain to their patients the importance of oral health.

Strong and significant evidence exists that diabetes has an impact on the prevalence and severity of periodontitis. Similarly, periodontitis contributes to the onset and persistence of hyperglycemia, poorer glycemic control increase in the incidence of periodontal disease [38, 39]. Thus, not only dental professionals but also physicians should educate their diabetic patients concerning the link between these two conditions and point out the bi-directional relationship between them. Certainly, there is an important gap in the education curricula concerning the correlation of oral to systemic health. In the present survey though, it was encouraging to observe that the majority of the participants expressed the willingness to learn how to screen their diabetic patients for periodontal disease.

The European Federation of Periodontology (EFP) and the International Diabetes Federation (IDF) published consensus guidelines for physicians, oral health professionals and patients for how to improve the early diagnosis, prevention and joint management of diabetes and periodontitis. The authors emphasize that the oral health care team has an important role in identifying pre-diabetes and undiagnosed diabetes mellitus. Similarly, endocrinologists, general health practitioners and other physicians need to be aware of the role of periodontal disease on the glycemic control and complications in people with diabetes [19].

## Conclusion

In conclusion, the overall knowledge of doctors about periodontitis was generally good. Some differences between endocrinologists and general physicians were depicted regarding their practice behaviors towards oral health during consultation. The lack of information during their training and the lack of collaboration with dentists could and should be improved to optimize the overall management of diabetic patients. A two-way professional relationship should be established.

## Data Availability

All data generated or analysed during this study are included in this published article.The data that support the findings of this study are available from the Division of Regenerative Dental Medicine and Periodontology, University Clinics of Dental Medicine, Faculty of Medicine, University of Geneva, Switzerland. Data are available from the author Alkisti ZEKERIDOU (email:alkisti.zekeridou@unige.ch) upon reasonable request and with permission of the University Clinics of Dental Medicine, Faculty of Medicine, University of Geneva.
